# Importing pancreata for transplantation: a single-center experience across evolving allocation eras

**DOI:** 10.3389/frtra.2025.1698617

**Published:** 2025-12-18

**Authors:** Riccardo Tamburrini, Stacey Hidalgo, Glen Leverson, Dixon B. Kaufman, Nikole A. Neidlinger, David P. Al-Adra, David D. Aufhauser, Carrie Thiessen, Didier Mandelbrot, Sandesh Parajuli, Jon S. Odorico

**Affiliations:** 1 University of Wisconsin School of Medicine and Public Health, Madison, WI, United States; 2 Division of Transplantation, Department of Surgery, University of Wisconsin School of Medicine and Public Health, Madison, WI, United States; 3 Department of Surgery, University of Wisconsin School of Medicine and Public Health, Madison, WI, United States; 4 UWHealth Transplant Center, Madison, WI, United States; 5 Division of Nephrology, Department of Medicine, University of Wisconsin School of Medicine and Public Health, Madison, WI, United States

**Keywords:** pancreas transplantation, organ allocation, procurement distance, cold ischemia time, graft survival

## Abstract

Recent changes to allocation systems have increased the geographic distribution of pancreas offers, often originating from outside a transplant center's donor service area or region. The impact of this wider sharing on outcomes remains uncertain. This study analyzed outcomes of primary pancreas transplants (2000–2018) at a large transplant center, stratified retrospectively on the nautical miles distance from the donor hospital. Primary endpoints were death-censored graft survival (DC-GS), patient survival, and graft thrombosis at different time points. No significant differences were found in DC-GS or patient survival for recipients of simultaneous pancreas-kidney (SPK), pancreas after kidney (PAK), or pancreas transplant alone (PTA), regardless of the distance from the donor hospital to the transplant center. Thrombosis rates were comparable across groups. Imported pancreata were from younger donors with lower BMI compared to locally recovered grafts. These findings support the notion that importing pancreata for transplantation is a feasible and safe practice that benefits patients, increases organ utilization, while benefiting transplant center volume data and reducing waiting times for patients. Encouraging wider importation may reduce waiting times and improve access to pancreas transplantation.

## Introduction

Pancreas transplantation is an effective and established treatment for Type 1 diabetes mellitus (T1DM), with or without end-stage renal disease (ESRD), and it is now offered more frequently to patients with Type 2 diabetes (T2DM) and ESRD (1–3). The procedure can establish optimal and durable glycemic control and is associated with increased life expectancy. It also prevents or stabilizes related complications such as nephropathy, neuropathy, and retinopathy (4). However, the number of available pancreata for transplant falls short of the demand. The transplant community is continually seeking strategies to extend the pancreatic donor pool, eliminate barriers impeding transplantation in certain regions and improve access to pancreas transplantation. There is heterogeneous acceptance and utilization of pancreata in the transplant community and therefore a transplantable pancreas may be declined by some centers but accepted by others. With increasing efforts to expand the donor pool, some centers have endeavored to importing more pancreatic allografts (5–10).

On March 2021, the Organ Procurement and Transplantation Network (OPTN) implemented a policy to remove OPTN region and donation service area (DSA) from kidney and pancreas allocation, with the goal of utilizing more pancreata by facilitating pancreas offers across regions and DSAs. The newly implemented allocation system replaced DSA as the first unit of distribution with a 250 nautical miles (NM) circle around the donor hospital (11). The policy changes did not result in increased pancreas transplants: the number of solitary pancreata transplanted declined from 143 in 2021 to 108 in 2022 while 820 SPK transplants were performed in 2021 and 810 in 2022. One factor potentially accounting for this is the reluctance to import pancreata from other regions due to logistical, economic, and psychological/trust limitations related to concerns with quality and the capability of unfamiliar recovery teams and practices (12–14).

In this paper, summarizing two decades of pancreas transplant activity, we present our experience and practice principles regarding pancreas transplantation across varying geographic distances. These data aim to assess the feasibility, safety, and limitations of broader pancreas allocation by analyzing transplant outcomes based on nautical mile (NM) distance from the donor hospital to the transplant center, reflecting the impact of the current allocation system.

## Methods

We performed a single-center retrospective study examining outcomes in all primary pancreatic transplants (PTX) performed between January 2000 and December 2018. We reviewed prospectively collected data from the University of Wisconsin Transplant Database.

### Patient population

The analysis of primary pancreatic transplants was conducted on all pancreas transplants grouped together and separately on simultaneous kidney-pancreas transplant (SPK), pancreas transplant alone (PTA) and pancreas-after kidney transplant (PAK) cohorts. Patients who underwent previous pancreas transplantation were excluded from this study. Donor and recipient clinical characteristics were obtained from UWHealth medical record after approval by the Institutional Review Board.

The data collected was analyzed based on the nautical mile (NM) distance from the donor hospital to the UW Health Transplant Center. Distances were retrospectively calculated and categorized into three groups: (i) less than 250 NM, (ii) between 250 and 750 NM, and (iii) greater than 750 NM. We assessed the association between procurement distance and several outcomes of interest.

### Outcomes of interest, variables and definitions

Death-censored pancreas (DC-GS) graft survival was reported at 90-days and 5-years post-transplant. Patient survival was reported at 5-years post-transplant. Post-transplant pancreatectomy and graft-thrombosis are respectively reported at 30 and 90-days post-transplant. These outcomes were investigated and analyzed among all transplants and according to transplant type and distance in NM from the transplant center. DC-Graft failure was defined by the current UNOS definition of one or more of the following: pancreatectomy, re-listing or re-transplantation of pancreas or islets, or insulin use >0.5 U/kg/day (1).

### Patient selection and evaluation

Patients considered for PTX included selected patients with ESRD and insulin-dependent diabetes, both T1DM and T2DM (SPK or PAK), and patients with T1DM and impaired awareness of hypoglycemic events or frequent severe hypoglycemic events despite optimal care by an endocrinologist (PTA). All patients undergoing transplantation were approved by the multi-disciplinary patient listing committee.

### Operative technique

Surgical techniques were consistent throughout the study period as previously described (15). Pancreaticoduodenal allografts were placed head up with enteric drainage of exocrine secretions via the mid to proximal jejunum and systemic venous drainage of endocrine secretions into the iliac venous system, via a midline incision. The majority of pancreatic and renal allografts were placed in the right and left iliac fossa respectively; some SPK and PAK grafts were implanted ipsilaterally (16).

### Clinical management and immunosuppression

Pre-transplant immunological risk and desire for a steroid-free regimen influenced the selection of an individualized immunosuppressive regimen. Induction therapy included alemtuzumab, anti-thymocyte globulin or IL-2 receptor antagonists (basiliximab or daclizumab), with IL-2 receptor antagonists preferred for SPK transplants and T-cell depleting agents for solitary pancreas transplants (17). Tacrolimus and oral mycophenolic acid were initiated as maintenance immunosuppressive therapy.

### Statistical analysis

Categorical variables were summarized by reporting counts (percentages) and compared between groups using Fisher's exact tests, and chi-square tests, alternatively. Continuous variables were reported using means ± standard deviations and compared using analysis of variance (ANOVA). Kaplan–Meier rates of DC-GS, patient survival, post-transplant pancreatectomy, and thrombosis were reported at 30-days, 90-days, or 5-years and overall survival was compared using a log-rank test. Statistical significance was set at *p* < 0.05. All analyses were performed using SAS version 9.4 (SAS Institute, Inc., Cary, NC).

## Results

Throughout the study period between January 2000 and December 2018, a total of 884 primary pancreas transplants were performed. Of these, 709 grafts (80%) were recovered from hospitals within 250 nautical miles (NM), 102 (11%) were recovered from a distance of 250 to 750 NM, and 73 (8%) were procured from more than 750 NM away. Among all pancreas transplants, 676 were simultaneous pancreas-kidney (SPK, 77%), 152 were pancreas transplant alone (PTA, 17%), and 56 were pancreas after kidney (PAK, 6%). The distribution of grafts across NM distance categories for each transplant type is shown in Table 1.

**Table 1:** Number of transplanted pancreata by graft type and nautical mile distance from the donor hospital to the transplant center.

Primary pancreas transplant distribution by type and nautical miles (graphic)	Type of pancreas transplant	Overall cohort	Nautical miles analyses		
		Total	<250 NM	250–750 NM	>750 NM
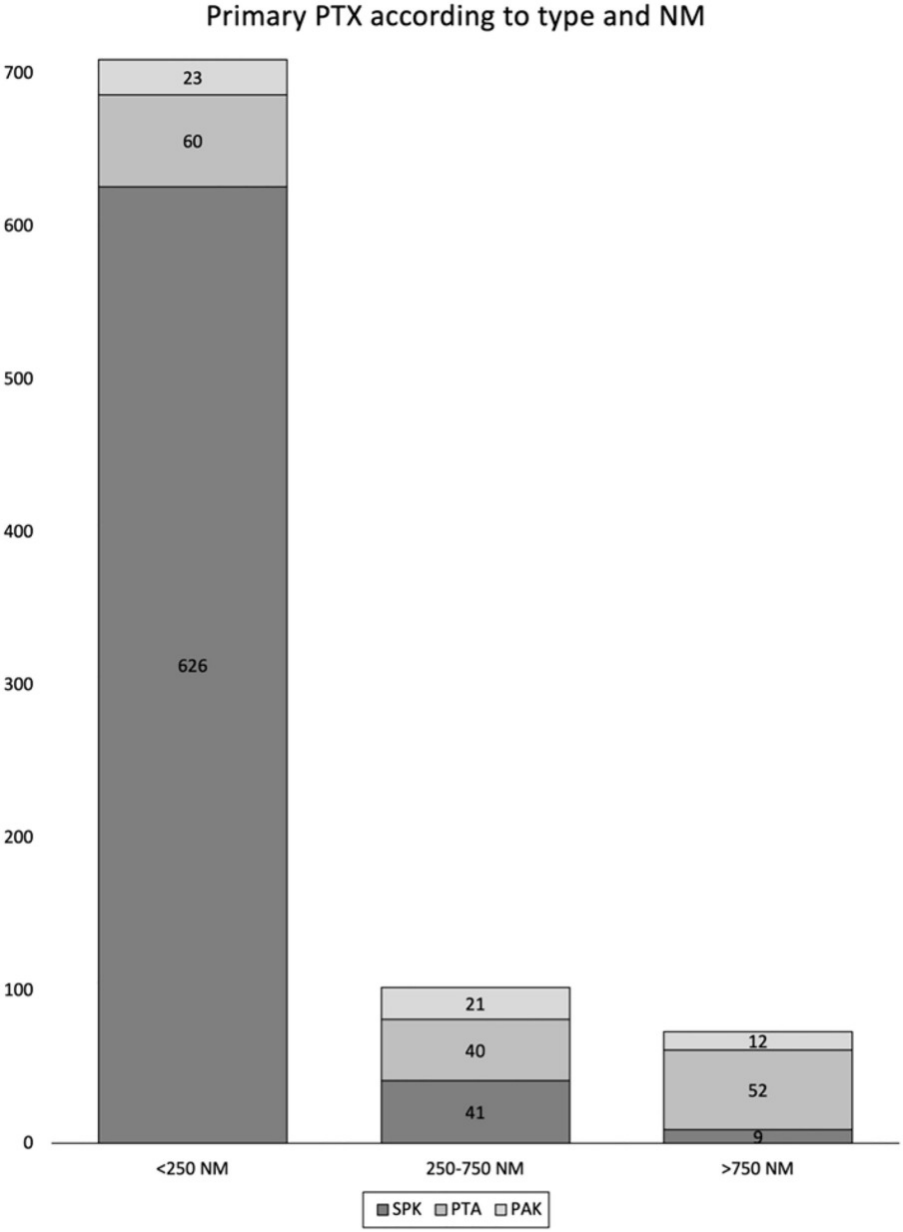	SPK	676 (77%)	626 (71%)	41 (5%)	9 (1%)
	PTA	152 (17%)	60 (4%)	40 (4%)	52 (6%)
	PAK	56 (6%)	23 (3%)	21 (2%)	12 (1%)
	All PTX	884 (100%)	709 (81%)	102 (11%)	73 (8%)

SPK, simultaneous pancreas-kidney transplant; PTA, pancreas transplant alone; PAK, pancreas after kidney transplant; PTX, pancreas transplant; NM, nautical mile.

### Patient survival, graft survival, graft thrombosis and graft pancreatectomy

We evaluated outcomes of interest in relation to the distance between donor hospital and transplant center.

#### All pancreatic transplants

**Figure 1:**
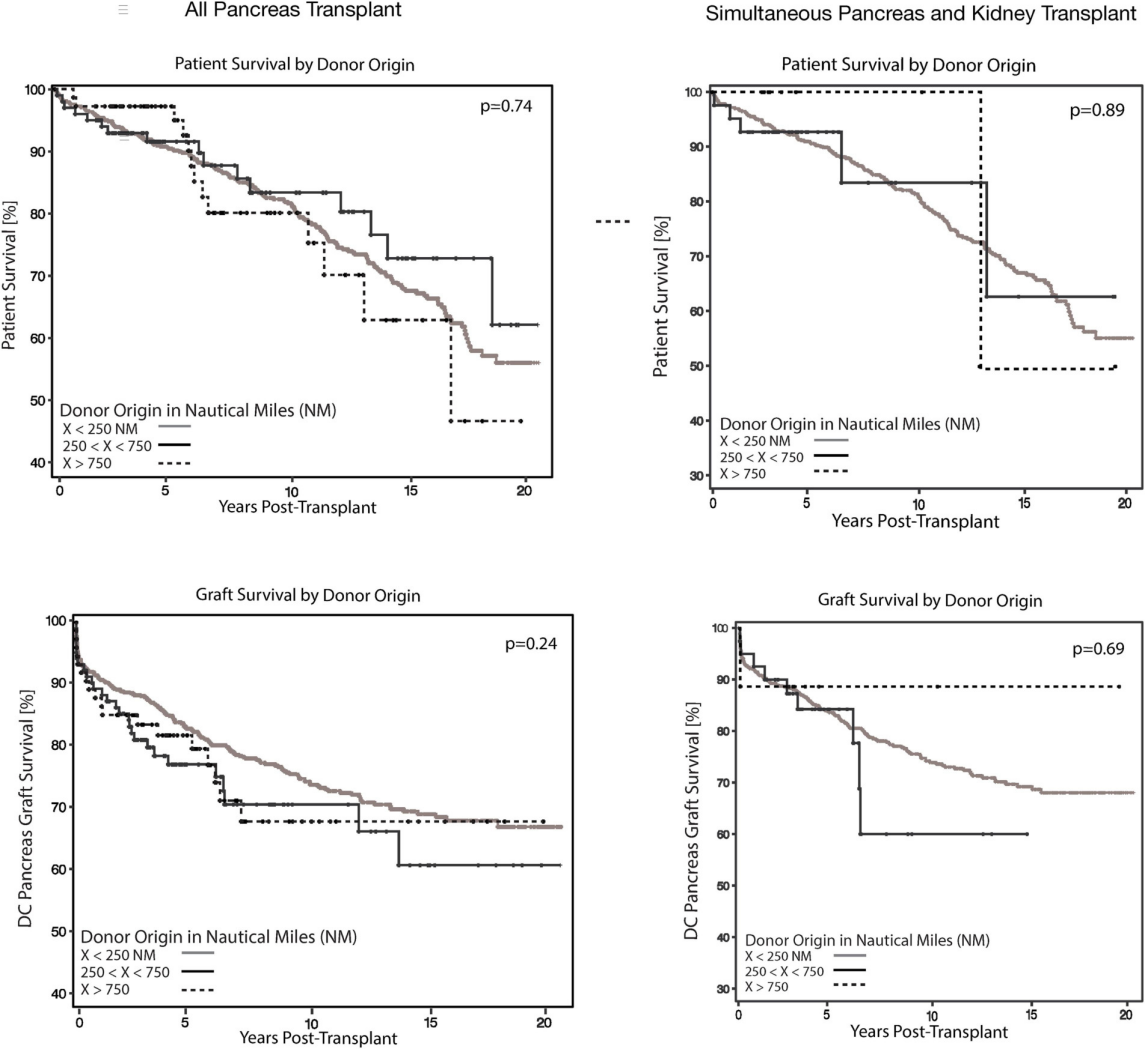
Patient and death-censored graft survival stratified by donor source for all pancreatic transplants and SPK transplants. Kaplan–Meier estimate of overall patient and death-censored graft survival stratified by donor source of all primary pancreatic transplants and simultaneous pancreas and kidney transplants (SPK) recipients. Neither patient nor death-censored graft survival showed differences associated with the donor origin at 5 years post-transplant.

**Figure 2:**
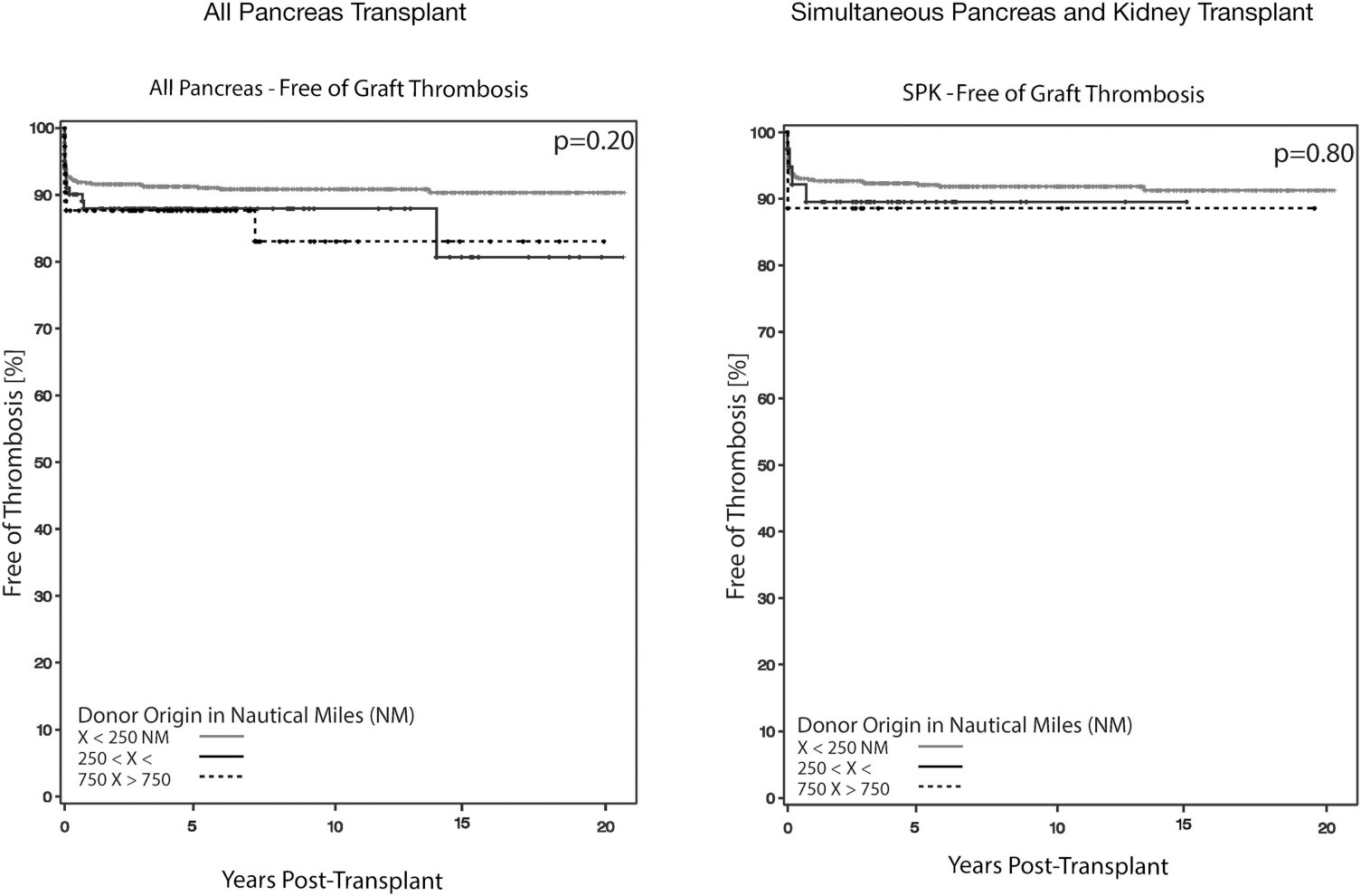
Rate of graft thrombosis for all primary pancreatic transplants and simultaneous pancreas and kidney transplants. Kaplan–Meier estimate of overall thrombosis graft rate for all primary pancreas transplants and simultaneous pancreas and kidney transplant for the period 2000–2018 stratified by nautical mile distance. There were no difference in graft thrombosis rate at 5-years post-transplant.

Major endpoints of the study are shown in Table 2. Evaluating all pancreatic transplants in the cohort, we found that the 90-day DC-GS rates were similar across all NM distance groups (<250 NM 95%, 250–750 NM 93%, >750 NM 92%; *p* = 0.24) (Table 2, Figure 1). Comparable outcomes were observed for 5-year DC-GS (<250 NM 82% vs. 250–750 NM 77% vs. >750 NM 79%; *p* = 0.24) and for 5-year patient survival (<250 NM 91% vs. 250–750 NM 92% vs. >750 NM 100%; *p* = 0.74). Pancreatectomy rate at 30 days post-transplant was not associated with NM distance (*p* = 0.80). Similarly, graft thrombosis rates at 90 days were comparable among the three NM groups (Table 2, Figure 2). Length of hospital stay (LOS) decreased progressively with increasing procurement distance (<250 NM 10.9 ± 7.1 days vs. 250–750 NM 9.5 ± 6.1 days vs. >750 NM 7.9 ± 3.3 days; *p* = 0.0005).

**Table 2:** Major transplant related endpoints of all pancreatic transplants.

Transplant-related endpoints	Nautical miles groups			
All pancreas transplant	<250 NM	250 NM–750 NM	>750 NM	*p*-value
Pancreas Graft Survival at 90 days (%)	95	93	92	0.24
Pancreas Graft Survival at 5 years (%)	82	77	79	0.24
Patient Survival at 5 years (%)	91	92	100	0.74
Pancreatectomy at 30 days (N.)	35	6	4	0.80
Graft Thrombosis at 90 days (%)	8	10	12	0.20
Thrombosis at Graft Loss (%)	3	5	5	0.52
Length of Hospital Stay	10.9 ± 7.1	9.5 ± 6.1	7.9 ± 3.3	0.0005
SPK Transplants				
Pancreas Graft Survival at 90 days (%)	93	95	89	0.69
Pancreas Graft Survival at 5 years (%)	83	84	89	0.69
Kidney Graft Survival at 5 years (%)	90	90	100	0.81
Delayed Graft Function (% grafts)	9	7	11	0.81
Patient Survival at 5 years (%)	91	93	100	0.89
Pancreatectomy at 30 days (*N*.)	28	1	1	0.41
Graft Thrombosis at 90 days (%)	6	8	11	0.80
Thrombosis at Graft Loss (%)	3	0	11	0.22
Length of Hospital Stay	11.2 ± 7.3	10.6 ± 7.2	10.9 ± 4.5	0.89
PAK Transplants				
Pancreas Graft Survival at 90 days (%)	100	86	75	0.66
Pancreas Graft Survival at 5 years (%)	76	70	67	0.65
Patient Survival at 5 years (%)	83	76	92	0.21
Pancreatectomy at 30 days (*N*.)	0	3	1	0.14
Graft Thrombosis at 90 days (%)	9	14	17	0.81
Thrombosis at Graft Loss (%)	0	14	8	0.46
Length of Hospital Stay	9.8 ± 4.7	11.7 ± 6.9	9.7 ± 5.0	0.48
PTA Transplants				
Pancreas Graft Survival at 90 days (%)	88	95	96	0.79
Pancreas Graft Survival at 5 years (%)	71	73	81	0.79
Patient Survival at 5 years (%)	95	100	98	0.33
Pancreatectomy at 30 days (*N*.)	7	2	2	0.28
Graft Thrombosis at 90 days (%)	20	10	12	0.50
Thrombosis at Graft Loss (%)	8	5	4	0.77
Length of Hospital Stay	8.5 ± 4.9	7.2 ± 3.1	7.0 ± 1.9	0.07

SPK, simultaneous pancreas-kidney transplants; PAK, pancreas after kidney transplants; PTA, pancreas transplants alone.

#### Simultaneous pancreas and kidney transplants

Given the inherent differences between SPK and solitary pancreas transplants, outcomes were also analyzed separately. In SPK recipients, NM distance had no significant impact on DC-GS at either 90 days (Table 2, *p* = 0.69, Figure 1) or 5 years (*p* = 0.69). Patient survival rates at 5 years were also comparable (*p* = 0.89). Neither pancreatectomy within 30 days (*p* = 0.41) nor graft thrombosis at 90 days (*p* = 0.80) were influenced by procurement distance (Table 2, Figure 2). LOS and the incidence of kidney delayed graft function were also similar across NM distance categories.

#### Pancreas-after-kidney and pancreas-alone transplants

**Figure 3:**
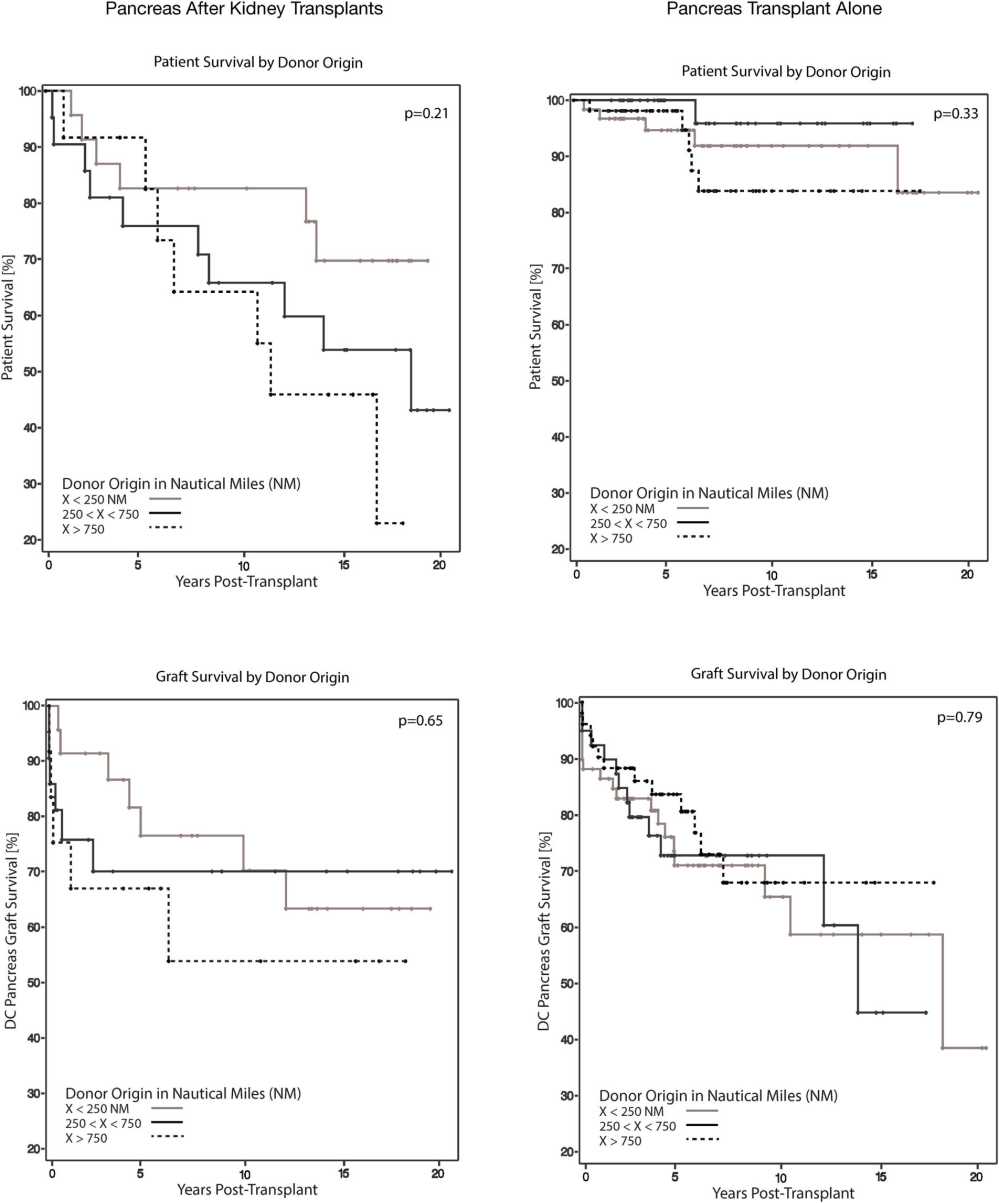
Patient and death-censored graft survival stratified by donor source for PAK and PTA transplants. Kaplan–Meier estimate of overall patient and death-censored graft survival stratified by donor source for pancreas after kidney (PAK) and pancreas transplant alone (PTA) recipients. Neither patient nor death-censored graft survival showed differences associated with the donor origin at 5 years post-transplant.

Among PAK and PTA recipients, NM distance did not influence 90-day or 5-year DC-GS (Table 2, Figure 3). Likewise, pancreatectomy within 30 days and graft thrombosis at 90 days post-transplant were not associated with donor hospital distance (Table 2).

### Donor demographics

Donor characteristics were analyzed according to NM distance from the transplant center (Table 3). Overall, 765 (87%) donations were from DBD donors and 119 (14%) from DCD donors, with no significant differences across NM distance categories (*p* = 0.34). Grafts procured from greater distances were from younger donors (*p* = 0.004) and with lower BMI (*p* = 0.0001). Cold ischemic time increased with greater distance (<250 NM 13.5 ± 4.5 h vs. 250–750 NM 16.4 ± 4.2 h vs. >750 NM 18 ± 2.8 h; *p* < 0.0001). PDRI was similar across groups (1.31 ± 0.52 vs. 1.34 ± 0.44 vs. 1.26 ± 0.41; *p* = ns), whereas KDPI was significantly lower for donors within 250 NM (22.3 ± 18.3 vs. 32.5 ± 20.4 vs. 23.9 ± 17.3%; *p* = 0.003). Donor demographic data were also analyzed separately by transplant type—SPK (Table 4), PAK (Supplementary Table S1), and PTA (Supplementary Table S2).

**Table 3:** Donor characteristics for All pancreas transplants.

Demographic and clinical variables	Overall	Nautical miles groups			
	Total	<250 NM	250 NM–750 NM	>750 NM	*p*-value
Age, years (Mean ± SD)		29.7 ± 12.5	26.02 ± 14.1	26.3 ± 12.4	0.004
BMI (Mean ± SD)		24.4 ± 4.4	22.7 ± 4.4	22.9 ± 4.2	0.0001
Gender					0.84
Males (%)	531 (60%)	429 (61%)	59 (58%)	43 (59%)	
Females (%)	353 (40%)	280 (39%)	43 (42%)	30 (41%)	
Race					<0.0001
American Indian/Alaska Native	7 (0.8%)	4	3	0	
Asian	10 (1.1%)	7	1	2	
Black or African American	59 (6.7%)	25	20	14	
Hispanic	28 (3.2%)	14	4	10	
Native Hawaiian/Pacific Islander	1 (0.1%)	1	0	0	
White	766 (86.7%)	649	73	44	
Unknown	13 (1.5%)	9	1	3	
Type of Transplant					0.34
DBD (%)	765 (87%)	607 (86%)	90 (88%)	68 (93%)	
DCD (%)	119 (14%)	102 (14%)	12 (12%)	5 (7%)	
Pancreas CIT, hours (Mean ± SD)		13.5 ± 4.5	16.4 ± 3.7	18 ± 2.8	<0.0001
Distance, miles (Mean ± SD)		72.4 ± 58.3	522.5 ± 151.6	1,197 ± 410.1	<0.0001
PDRI (Mean ± SD)		1.31 ± 0.52	1.34 ± 0.44	1.26 ± 0.36	NS
KDPI (Mean ± SD)		22.3 ± 18.3	32.5 ± 20.4	23.9 ± 17.3	0.003
Cause of Death					<0.0001
Anoxia	209 (24%)	144 (20%)	37 (36%)	28 (38%)	
Cerebrovascular Disease/Stroke	179 (20%)	143 (20%)	20 (20%)	16 (22%)	
Head Trauma	460 (52%)	392 (55%)	42 (41%)	26 (36%)	
CNS Tumor	11 (1%)	10 (1%)	1 (1%)	0 (0%)	
Other Unspecified	25 (3%)	20 (3%)	2 (2%)	3 (4%)	

BMI, body mass index; DBD, donation after brain death; DCD, donation after circulatory death; CIT, cold ischemia time; SD, standard deviation.

**Table 4:** Donor characteristics for primary simultaneous pancreas and kidney transplants.

Demographic and clinical variables	Overall	Nautical miles groups			
	Total	<250 NM	250 NM–750 NM	>750 NM	*p*-value
Age, years (Mean ± SD)		29.7 ± 12.4	24.2 ± 15.1	29.55 ± 11.1	0.02
BMI (Mean ± SD)		24.4 ± 4.3	22.2 ± 4.2	21.9 ± 5.1	0.002
Gender					0.44
Males (%)	414 (61%)	387 (62%)	23 (56%)	4 (44%)	
Females (%)	262 (39%)	239 (38%)	18 (44%)	5 (56%)	
Race					<0.0001
American Indian/Alaska Native	5 (0.7%)	4	1	0	
Asian	6 (0.9%)	5	1	0	
Black or African American	29 (4.3%)	16	10	3	
Hispanic	12 (1.8%)	11	1	0	
Native Hawaiian/Pacific Islander	0 (0%)	0	0	0	
White	616 (91.1%)	582	28	6	
Unknown	8 (1.2%)	8	0	0	
Type of Transplant					0.32
DBD (%)	568 (84%)	527 (84%)	32 (78%)	9 (100%)	
DCD (%)	108 (16%)	99 (16%)	9 (22%)	0 (0%)	
Pancreas CIT, hours (Mean ± SD)		13.5 ± 4.4	16.7 ± 2.6	18 ± 2.3	<0.0001
Kidney CIT, hours (Mean ± SD)		14.69 ± 4.48	18.87 ± 2.38	20 ± 2.13	<0.0001
Distance, miles (Mean ± SD)		68 ± 56	513 ± 134	1,122 ± 234	<0.0001
PDRI (Mean ± SD)		1.32 ± 0.54	1.49 ± 0.52	1.36 ± 0.52	NS
KDPI (Mean ± SD)		22.3 ± 18.3	32.5 ± 20.4	23.9 ± 17.3	0.003
Cause of Death					<0.0001
Anoxia	145 (21%)	121 (19%)	20 (49%)	4 (44%)	
Cerebrovascular Disease/Stroke	132 (20%)	123 (20%)	7 (17%)	2 (22%)	
Head Trauma	371 (55%)	357 (57%)	13 (32%)	1 (11%)	
CNS Tumor	8 (1%)	8 (1%)	0 (0%)	0 (0%)	
Other Unspecified	20 (3%)	17 (3%)	1 (2%)	2 (22%)	

BMI, body mass index; DBD, donation after brain death; DCD, donation after circulatory death; CIT, cold ischemia time; SD, standard deviation.

### Recipient demographics

Recipient characteristics according to NM distance are presented in Table 5. Overall, T1DM was the predominant indication for pancreas transplantation (*n* = 824, 93%), followed by T2DM (*n* = 50, 6%) and other causes (*n* = 10, 1%). Most SPK recipients received IL-2 receptor antagonists for induction, whereas solitary pancreas recipients more frequently received T-cell depletion, reflecting their higher immunologic risk (Table 5). The panel-reactive antibody (PRA) level was significantly lower in recipients of grafts procured within 250 NM compared to those from greater distances (<250 NM 5.01 ± 16.03% vs. 250–750 NM 11.96 ± 26.13% vs. >750 NM 11.37 ± 23.77%; *p* < 0.0003). Recipient demographic data were also analyzed separately by transplant type: SPK (Table 6), PAK (Supplementary Table S3), and PTA (Supplementary Table S4).

**Table 5:** Recipient characteristics for all pancreas transplants.

Demographic and clinical variables	Overall	Nautical miles groups			
	Total	<250 NM	250 NM–750 NM	>750 NM	*p*-value
Age, years (Mean ± SD)		42.0 ± 8.7	43.7 ± 9.5	43.8 ± 10.1	0.08
BMI (Mean ± SD)		25.5 ± 3.9	26.5 ± 3.4	26.4 ± 3.5	0.009
Gender					0.28
Males (%)	522 (59%)	426 (60%)	59 (58%)	37 (51%)	
Females (%)	362 (41%)	283 (40%)	43 (42%)	36 (49%)	
Race					0.45
American Indian/Alaska Native	7 (0.8%)	7	0	0	
Asian	14 (1.6%)	13	0	1	
Black or African American	56 (6.3%)	47	7	2	
Native Hawaiian/Pacific Islander	4 (0.5%)	4	0	0	
White	802 (90.7%)	638	94	70	
Declines to answer	1 (0.1%)	0	1	0	
Indication for Transplant					0.08
Diabetes Mellitus—Type I	824 (93.2%)	663 (93%)	94 (92%)	67 (92%)	
Diabetes Mellitus—Type II	50 (5.7%)	40 (6%)	6 (6%)	4 (5%)	
Diabetes Mellitus—Type Other/Unknown	8 (0.9%)	6 (1%)	0 (0%)	2 (3%)	
Diabetes Secondary to chronic Pancreatitis	2 (0.2%)	0 (0%)	2 (2%)	0 (0%)	
Diabetes Duration (Months ± SD)		26.7 ± 8.9	27.7 ± 10.0	28.2 ± 9.7	NS
Induction					<0.0001
ATG and Thymoglobulin	180 (20.4%)	110 (15.5%)	38 (37.3%)	32 (43.8%)	
Campath	276 (31.2%)	223 (31.5%)	31 (30.4%)	22 (30.1%)	
Rituximab	1 (0.1%)	1 (0.1%)	0 (0%)	0 (0%)	
IL-2 receptor antagonist	427 (48.3%)	375 (52.9%)	33 (32.4%)	19 (26.0%)	
PRA (% Mean ± SD)		5.01 ± 16.03	11.96 ± 26.13	11.37 ± 23.77	0.0003

BMI, body mass index; DBD, donation after brain death; DCD, donation after circulatory death; cold ischemia time; SD, standard deviation.

**Table 6:** Recipient characteristics for simultaneous pancreas-kidney transplants.

Demographic and clinical variables	Overall	Nautical miles groups			
	Total	<250 NM	250 NM–750 NM	>750 NM	*p*-value
Age, years (Mean ± SD)		41.9 ± 8.4	42.5 ± 8.8	43.9 ± 10.7	0.69
BMI (Mean ± SD)		25.3 ± 3.8	25.6 ± 3.2	26.9 ± 4.5	0.27
Gender					0.68
Males (%)	419 (62%)	386 (62%)	26 (63%)	37 (84%)	
Females (%)	257 (38%)	240 (38%)	15 (37%)	7 (16%)	
Race					0.29
American Indian/Alaska Native	7 (1%)	7	0	0	
Asian	12 (1.8%)	12	0	0	
Black or African American	55 (8.1%)	46	7	2	
Native Hawaiian/Pacific Islander	4 (0.6%)	4	0	0	
White	598 (88.5%)	557	34	7	
Declines to answer	0 (0%)	0	1	0	
Indication for Transplant					0.03
Diabetes Mellitus—Type I	624 (92.3%)	583 (93%)	35 (85%)	6 (67%)	
Diabetes Mellitus—Type II	49 (7.2%)	40 (6%)	6 (15%)	3 (33%)	
Diabetes Mellitus—Type Other/Unknown	2 (0.3%)	2 (<1%)	0 (0%)	0 (0%)	
Diabetes Secondary to chronic Pancreatitis	1 (0.1%)	1 (<1%)	0 (0%)	0 (0%)	
Diabetes Duration (Months ± SD)		26.4 ± 8.7	29.2 ± 10.7	27 ± 6.8	NS
Pre-Transplant Dialysis	497 (Y)	457 (73%)	33 (80%)	7 (78%)	0.59
	179 (*N*)	169 (27%)	8 (20%)	2 (22%)	
Delayed Kidney Graft Function	Y 61 (9%)	57 (9%)	3 (7%)	1 (11%)	0.81
	N 615 (91%)	569 (91%)	38 (93%)	8 (89%)	
Induction					<0.0001
ATG and Thymoglobulin	95 (14%)	79 (12.6%)	16 (39%)	2 (22.2%)	
Campath	212 (31.4%)	198 (31.5%)	10 (24%)	4 (44.4%)	
Rituximab	1 (0.1%)	1 (0.2%)	0 (0%)	0 (0%)	
IL-2 receptor antagonist	368 (54.4%)	350 (55.7%)	15 (37%)	3 (33.3%)	
PRA (% Mean ± SD)		4.05 ± 14.1	10.54 ± 24.85	3.44 ± 9.23	0.03

BMI, body mass index; donation after brain death; DCD, donation after circulatory death; CIT, cold ischemia time; SD, standard deviation.

## Discussion

In an era of evolving pancreas allocation policies and persistent underutilization of pancreas grafts, the question of whether pancreata can be safely and effectively imported remains both timely and clinically significant. In 2021, the OPTN replaced donation service areas and regions with a concentric-circle distribution model, establishing 250 nautical miles (NM) around the donor hospital as the first unit of allocation (11). This change was intended to promote equity and broader sharing, but it also increased the need to import grafts and rely on distant recovery teams. Many transplant centers remain hesitant to expand importation because of concerns about longer cold ischemia times (CIT), uncertainty about donor team expertise, and the logistical complexity of transportation and communication. The present study addresses these concerns directly by analyzing nearly two decades of pancreas transplant experience at a high-volume center, providing one of the most detailed single-center assessments of distance-based pancreas importation to date.

Across 884 primary pancreas transplants performed between 2000 and 2018, outcomes remained comparable regardless of procurement distance. Ninety-day death-censored graft survival (DC-GS) was 95%, 93%, and 92% for grafts recovered within <250 NM, 250–750 NM, and >750 NM, respectively, with similar 5-year graft and patient survival rates. Early technical complications—including graft thrombosis and 30-day pancreatectomy—were not associated with distance, and length of hospital stay was slightly shorter among recipients of grafts recovered from farther distances. These findings suggest that, when appropriate donor selection and logistical coordination are in place, pancreas grafts can be imported safely—even from beyond 750 NM—without compromising clinical outcomes.

As expected, CIT increased with greater procurement distance, ranging from 13.5 h for locally recovered grafts to 18 h for grafts from beyond 750 NM. Within this range, CIT did not correlate with poorer graft or patient outcomes. This likely reflects both improved preservation methods and a tendency to import higher-quality grafts. Indeed, imported pancreata in this cohort were generally from younger donors with lower BMI, indicating selective acceptance that may have offset the impact of longer ischemia. While we did not perform multivariable analyses, these descriptive trends reinforce that distance alone is not a determinant of outcome when donor quality and recovery processes are optimized. Interpretation of our findings should therefore focus on feasibility and safety within a context of selective importation, rather than statistical equivalence across all donor types.

A successful pancreas transplant outcome depends on many factors, but preeminent among these are donor factors, such as the quality of the donor, the expertise of the recovery surgeon and the cold ischemia time, factors which if optimal can set the overall recovery of the recipient on a positive trajectory, or instead if suboptimal, on a complicated trajectory. A goal of this new allocation system was to promote greater regional and national sharing of organs, which in turn necessitates importing more organs and relying on other recovery teams to perform organ recoveries and assess organ quality. Because importing organs raises the logistical complexities around organ recovery, particularly related to transportation and communication, many transplant programs have not felt comfortable importing pancreata. The potential for increased CIT, and the lack of trust in the donor team for the quality of the recovery and their assessment of the pancreas combine to significantly raise the stakes for potentially poor outcomes in an already challenging type of transplant.

In this study we analyze our longstanding pre-COVID19 pandemic experience with importing pancreata, in order to assess the outcomes and potentially allay fears related to this practice and thereby help encourage the utilization of more transplantable pancreata. We analyzed 18 years of importing pancreata, using almost entirely local recovery teams and stratified transplants according to distance from the donor hospital to our center reflecting the newest changes in UNOS allocation policies. As the quality of the donor and donor recovery may have the greatest impact on ischemia reperfusion related complications and early technical failures, we focused on short-term surgical outcomes, including 90-day death-censored pancreas graft survival rates and the rate of pancreatectomy at 30 days and the 90-day rate of graft thrombosis, both partial and complete. We did not detect statistically significant differences in outcomes across donor source locations. While formal equivalence was not tested, the results suggest that increasing pancreas graft importation may be feasible when appropriate logistical and clinical resources are in place.

Whether the importation of pancreatic grafts is a safe practice is an important question, especially following recent allocation policy changes and future deployment of continuous distribution algorithms. This question has been addressed by several transplant groups, with generally favorable results (5–10, 12). Fridell et al. found no differences in graft survival between local and imported pancreatic grafts (63 of 274) that were previously refused by other transplant centers (5), they also reported no differences in graft and patient survival regardless of the procuring team. Torabi et al. showed that increasing the import rate of pancreatic grafts did not compromise transplant outcomes in terms of graft and patient survival and did not increase hospital resource expenditures in terms of hospital stay (8). Finger et al. published the largest analysis comparing outcomes of locally procured and imported grafts. They reported on 1,014 cases from 1998 to 2008 and showed that importation had no effect on recipient or graft survival in solitary pancreas transplants (12). They also observed no statistically significant effect on technical failure rates, or long-term graft or patient survival in SPK recipients. However, imported pancreata were not stratified by region or beyond region, and the cohort included 226 retransplants, 674 BD pancreas transplants, and unequal number of DCD donors in each arm, thus limiting the interpretability of the study. Moreover, technical failure rates were numerically higher in imports: SPK (17.6 vs. 12.2%), PTA (11.3 vs. 8.8%) and PAK (9.7 vs. 4.9%) transplants, and more early deaths occurred in imported graft recipients (34/705, 4.8%) than in local graft recipients (9/309, 2.9%). Choinski et al. reported that importation was associated with significantly shorter WL times (−48%) for SPK in Region 9, but with 37% higher mean charges (18). Owen-Simon et al. recently reported outcomes of 81 pancreas transplants, of which 62 were locally procured and 19 were imported from areas greater than 250 NM from their center. They reported that recipients of imported grafts received grafts from younger donors having a lower BMI; additionally, they report a reduction of the waiting times since the practice of importing pancreata was introduced (14). Booker et al. recently analyzed OPTN data for SPK and PA transplants performed in the 2 years before and 2 years after the UNOS policy change involving the removal of DSA and OPTN regions from allocation algorithms. They reported that this policy change resulted in broader distribution with minimal impact on short-term clinical outcomes (13). Adler et al. reported on 199 solitary pancreas grafts of which 184 were imported from another DSA with a median match rank of 49 (interquartile range 14–129) indicating the accepting offers independent of prior centers' decisions can result in quality utilization of imported pancreata and shorten waiting times for patients (10). Taken together these results support the feasibility of importing pancreata for transplantation.

Here, we report results of a large series of imported pancreata. We chose to stop inclusion by 2018 so that we can achieve a minimum of 5-year follow-up for all patients. Overall graft and patient survival rates did not differ significantly among groups when analyzed by donor hospital location, distance from the transplant center, or donor DBD/DCD status. Similarly, while formal equivalence testing was not performed, no statistically significant differences were observed in technical failure rates, whether defined as pancreatectomy within 30 days or graft thrombosis within 90 days post-transplant. Our experience underscores that the key to safe importation lies in communication and consistency. The majority of imported grafts in this series were procured by local recovery teams, and our center implemented structured protocols to ensure thorough graft evaluation. These included direct discussions with the procuring surgeon, review of *in situ* and back-table photographs, detailed anatomical descriptions, and, whenever possible, real-time video assessment of the graft ex vivo. This approach reduced uncertainty and enabled our center to maintain a discard rate below 5% in recent years. Grafts that were ultimately not used were most often declined due to intraoperative findings such as vascular injury or parenchymal compromise, rather than prolonged transport time.

This study is notable for several key innovations. First, it presents one of the largest single-center series of pancreas transplants to date, with 884 primary deceased donor grafts and nearly two decades of follow-up. Second, unlike previous studies that broadly categorized grafts as either “local” or “imported,” we applied both historical UNOS regional designations and direct nautical mile (NM) stratification—an approach that mirrors the current OPTN concentric-circle allocation policy. By incorporating contemporary donor risk metrics (such as PDRI), adjusting for transplant type (SPK, PAK, PTA), and analyzing outcomes across granular distance thresholds, including >750 NM, our findings offer a detailed and policy-relevant assessment of pancreas graft importation. This granularity enables a more accurate appraisal of logistical feasibility and graft performance, especially in solitary pancreas transplants. Finally, the results have relevance beyond the U.S., offering data that may inform cross-border pancreas sharing frameworks in other healthcare systems seeking to expand equitable access to transplantation.

The limitations of this study include the nature of being a retrospective single-center study and as such conclusions must be interpreted with caution. We were unable to perform a formal cost analysis related to transportation and import of pancreata. However, there is no doubt that the typical air charter costs of $15–30,000 has increased the cost of goods. Performing a formal cost analysis of pancreatic graft transplantation presents several challenges due to the inherent complexity of the process. Logistical costs, including specialized transportation, can vary significantly depending on the distances involved, air charter and courier demand/availability, weather, geography, and types of transportation employed (air charter, commercial flight, ground transport). Furthermore, the availability of a local recovery teams or the need for transporting our team to recover the pancreas, can also impact overall costs. Limited local resources in remote areas may necessitate additional expenses for travel, coordination, and personnel time. Every case may be different, so a lack of standardized data on these diverse costs further hinders the ability to perform a comprehensive and accurate cost analysis for pancreatic graft transplantation.

In this manuscript, we are unable to calculate the exact rate of discarded pancreatic grafts after transportation. Nonetheless, we estimate that nowadays less than 5% of the grafts we import are not utilized for transplantation. The reasons for the non-utilization of imported pancreatic grafts are myriad and include: underappreciated procurement errors, unexpected findings during further assessment of the graft upon arrival, such as compromised vascular integrity or unexpected firmness, nodularity, fibrosis, parenchymal or duodenal damage, which may render the graft unsuitable for transplantation. Additionally, unforeseen logistical snafus, such as delays in transportation resulting in prolonged cold ischemia time can negatively impact graft viability and lead to discard after transportation. In rare cases, recipient-related factors, such as sudden changes in the patient's condition may also lead to the decision not to proceed with the transplantation of an imported graft. In an effort to minimize discards after transportation, in all cases nowadays, we attempt to speak with the donor surgeon prior to the recovery to go over the recovery procedural plan because there are so many variations in technique being practiced in the community. In addition, we prefer to receive both *in situ* and back table photos of the graft after back table additional flush of both arteries. In all cases we receive an anatomical description and verbal assessment by the recovery surgeon, and in most cases, we try to conduct a Face Time survey/tour of the pancreas graft ex vivo if feasible. Thus, we make every effort to minimize discards; however, the lack of detailed recorded data on this aspect limits our ability to provide a more in-depth analysis. Future research should aim to include such data to offer a more comprehensive understanding of graft non-utilization rates and their impact on transplantation costs and outcomes.

Furthermore, our data do not allow us to analyze the potential benefits of using local recovery teams. In the present study, the vast majority of imported pancreatic grafts were procured by local recovery teams. Due to the high transportation costs and personnel limitations, we have not generally utilized our own teams to recover nationally imported pancreata during this pre-pandemic series. Therefore, we cannot directly comment on the potential benefits of employing one's own recovery team. Nonetheless, we do believe there are strong benefits to sending one's team to recover regional pancreata beyond the transplant center DSA, but usually within a reasonable distance and depending on team availability. The potential benefits of sending one's own recovery team include more efficient communication, trusted recovery methods, efficient transportation, and opportunity for team training. Thus, our current practice is to send our team to recover the pancreas whenever feasible and practicable, but this does depend on distance, whether the donor is a DCD donor, availability of our recovery team and other anticipated quality indicators (donor age, BMI, PDRI, CT scan findings, etc.). While not absolute, we would consider sending our team up to 500 miles to recover the pancreas.

The screening process for organ acceptance can be challenging and not only related to donor demographic characteristics but also related to geographical position of the transplant center, availability of commercial flight or any other transportation, timing of the donor, availability and expertise of local procurement teams, costs of transportation, virtual crossmatch capabilities and most importantly effective communication between the local recovery surgeon/team and the transplant team with photographic material of the graft before and after flush with preservation solution (9, 10). There is no doubt that these real-world factors limit more widespread implementation of graft importation.

In conclusion, this study is the notion that importing pancreatic grafts to increase organ utilization is feasible and safe. Transplants involving grafts procured within 250 NM of our center achieved outcomes that were not statistically different from those involving grafts procured from beyond 250 NM, within the context of some degree of increased selectivity for imported organs. While this analysis was not designed to establish equivalence, the findings support the potential for pancreas graft importation to increase transplant rates and reduce waiting times for eligible recipients. We recognize that logistical and personnel challenges—including transportation costs, staffing limitations, and variability in local recovery expertise—remain barriers to broader implementation. Nonetheless, these data should encourage more pancreas graft importation and stimulate the deployment of transplant resources needed to manage this practice.

## Data Availability

The raw data supporting the conclusions of this article will be made available by the authors, without undue reservation.
