# Supplementary material for: Importing pancreata for transplantation: a single-center experience across evolving allocation eras
Source: Front Transplant. 2025 Dec 18;4:1698617. doi: 10.3389/frtra.2025.1698617 (PMC12756387; doi:10.3389/frtra.2025.1698617)
Supplement: Supplementary file 1 [file Table1.docx]

|  |  | **Nautical Miles Analyses** | | | |
| --- | --- | --- | --- | --- | --- |
|  | Total | <250 NM | 250NM-750NM | >750NM | p-value |
| Age, years (Mean ± SD) |  | 28.1 ± 12.6 | 26.6 ± 14.7 | 28.0 ± 11.3 | 0.92 |
| BMI (Mean ± SD) |  | 24.0 ± 5.2 | 24.1 ± 4.1 | 24.9 ± 4.4 | 0.84 |
|  |  |  |  |  |  |
| Gender |  |  |  |  | 0.82 |
| Males (%) | 35 | 15 (65%) | 12 (57%) | 8 (67%) |  |
| Females (%) | 21 | 8 (35%) | 9 (43%) | 4 (33%) |  |
|  |  |  |  |  |  |
| Race |  |  |  |  | 0.28 |
| American Indian/Alaska Native | 0 | 0 (0%) | 0 (0%) | 0 (0%) |  |
| Asian | 0 | 0 (0%) | 0 (0%) | 0 (0%) |  |
| Black or African American | 10 | 3 (13%) | 4 (19%) | 3 (25%) |  |
| Hispanic | 3 | 1 (4%) | 0 (0%) | 2 (17%) |  |
| Native Hawaiian/Pacific Islander | 1 | 1 (4%) | 0 (0%) | 0 (0%) |  |
| White | 40 | 18 (78%) | 16 (76%) | 6 (50%) |  |
| Unknown | 2 | 0 (0%) | 1 (5%) | 1 (8%) |  |
|  |  |  |  |  |  |
| Type of Transplant |  |  |  |  | 1 |
| DBD (%) | 55 | 22 (96%) | 21 (100%) | 12 (100%) |  |
| DCD (%) | 1 | 1 (4%) | 0 (0%) | 0 (0%) |  |
|  |  |  |  |  |  |
| Pancreas cold ischemic time, hours (Mean ± SD) |  | 15.7 ± 4.3 | 16.3 ± 5.1 | 20.1 ± 3.7 | 0.02 |
|  |  |  |  |  |  |
| Distance, miles (Mean ± SD) |  | 107 ± 69 | 484 ± 166 | 1283 ± 775 | <0.0001 |
| PDRI (Mean ± SD) |  | 1.26 ± 0.39 | 1.27 ± 0.43 | 1.23 ± 0.30 | NS |
|  |  |  |  |  |  |
| Cause of Death |  |  |  |  | 0.12 |
| Anoxia | 9 | 2 (9%) | 7 (33%) | 0 (0%) |  |
| Cerebrovascular Disease/Stroke | 13 | 6 (26%) | 3 (14%) | 4 (33%) |  |
| Head Trauma | 32 | 14 (31%) | 11 (52%) | 7 (58%) |  |
| CNS Tumor | 0 | 0 (0%) | 0 (0%) | 0 (0%) |  |
| Other Unspecified | 2 | 1 (4%) | 0 (0%) | 1 (8%) |  |

Supplemental Table 1: Donor Characteristics for Primary Pancreas After Kidney (PAK) Transplants. Body Mass Index (BMI), Donation after Brain Death (DBD), Donation after Circulatory Death (DCD), Cold Ischemia Time (CIT), Standard Deviation (SD)

|  |  | **Nautical Miles Groups** | | | |
| --- | --- | --- | --- | --- | --- |
|  | Total | <250 NM | 250NM-750NM | >750NM | p-value |
| Age, years (Mean ± SD) |  | 30.4 ± 13.6 | 27.5 ± 13.0 | 25.3 ± 12.8 | 0.13 |
| BMI (Mean ± SD) |  | 24.4 ± 5.3 | 22.5 ± 4.8 | 22.7 ± 4.0 | 0.07 |
|  |  |  |  |  |  |
| Gender |  |  |  |  | 0.21 |
| Males (%) | 82 | 27 (45%) | 24 (60%) | 31 (60%) |  |
| Females (%) | 70 | 33 (55%) | 16 (40%) | 21 (40%) |  |
|  |  |  |  |  |  |
| Race |  |  |  |  | 0.11 |
| American Indian/Alaska Native | 2 | 0 (0%) | 2 (5%) | 0 (0%) |  |
| Asian | 4 | 2 (3%) | 0 (0%) | 2 (4%) |  |
| Black or African American | 20 | 6 (10%) | 6 (15%) | 8 (15%) |  |
| Hispanic | 13 | 2 (3%) | 2 (8%) | 8 (15%) |  |
| Native Hawaiian/Pacific Islander | 0 | 0 (0%) | 0 (0%) | 0 (0%) |  |
| White | 110 | 49 (82%) | 29 (72%) | 32 (62%) |  |
| Unknown | 3 | 1 (2%) | 0 (0%) | 2 (4%) |  |
|  |  |  |  |  |  |
| Type of Transplant |  |  |  |  | 0.38 |
| DBD (%) | 142 | 58 (97%) | 37 (93) | 47 (90%) |  |
| DCD (%) | 10 | 2 (3%) | 3 (7%) | 5 (10%) |  |
|  |  |  |  |  |  |
| Pancreas cold ischemic time, hours (Mean ± SD) |  | 13.0 ± 4.7 | 16.1 ± 3.8 | 17.5 ± 2.4 | <0.0001 |
|  |  |  |  |  |  |
| Distance, miles (Mean ± SD) |  | 101 ± 67 | 552 ± 159 | 1190 ± 311 | <0.0001 |
| PDRI (Mean ± SD) |  | 1.25 ± 0.40 | 1.24 ± 0.31 | 1.26 ± 0.35 | NS |
|  |  |  |  |  |  |
| Cause of Death |  |  |  |  | 0.46 |
| Anoxia | 55 | 21 (35%) | 10 (25%) | 24 (46%) |  |
| Cerebrovascular Disease/Stroke | 34 | 14 (23%) | 10 (25%) | 10 (19%) |  |
| Head Trauma | 57 | 21 (35%) | 18 (45%) | 18 (35%) |  |
| CNS Tumor | 3 | 2 (3%) | 1 (2.5%) | 0 (0%) |  |
| Other Unspecified | 3 | 2 (3%) | 1 (2.5%) | 0 (0%) |  |

Supplemental Table 2: Donor Characteristics for Primary Pancreas Transplant Alone (PTA) Transplants. Body Mass Index (BMI), Donation after Brain Death (DBD), Donation after Circulatory Death (DCD), Cold Ischemia Time (CIT), Standard Deviation (SD)

|  |  | **Nautical Miles Groups** | | | |
| --- | --- | --- | --- | --- | --- |
|  | Total | <250 NM | 250NM-750NM | >750NM | p-value |
| Age, years (Mean ± SD) |  | 47.9 ± 8.5 | 45.4 ± 8.9 | 46.3 ± 6.9 | 0.58 |
| BMI (Mean ± SD) |  | 27.03 ± 4.5 | 27.2 ± 3.3 | 27.2 ± 3.0 | 0.99 |
|  |  |  |  |  |  |
| Gender |  |  |  |  | 0.35 |
| Males (%) | 35 | 17 (74%) | 11 (52%) | 7 (58%) |  |
| Females (%) | 21 | 6 (26%) | 10 (48%) | 5 (42%) |  |
|  |  |  |  |  |  |
| Race |  |  |  |  | 0.38 |
| American Indian/Alaska Native | 0 | 0 (0%) | 0 (0%) | 0 (0%) |  |
| Asian | 1 | 0 (0%) | 0 (0%) | 1 (8%) |  |
| Black or African American | 1 | 1 (4%) | 0 (0%) | 0 (0%) |  |
| Native Hawaiian/Pacific Islander | 0 | 0 (0%) | 0 (0%) | 0 (0%) |  |
| White | 54 | 22 (96%) | 21 (100%) | 11 (92%) |  |
| Declines to answer | 0 | 0 (0%) | 0 (0%) | 0 (0%) |  |
|  |  |  |  |  |  |
| Indication for Transplant |  |  |  |  | 0.21 |
| Diabetes Mellitus – Type I | 55 | 23 (100%) | 21 (100%) | 11 (92%) |  |
| Diabetes Mellitus – Type II | 1 | 0 (0%) | 0 (0%) | 1 (8%) |  |
| Diabetes Mellitus – Type Other/Unknown | 0 | 0 (0%) | 0 (0%) | 0 (0%) |  |
| Diabetes Secondary to chronic Pancreatitis | 0 | 0 (0%) | 0 (0%) | 0 (0%) |  |
|  |  |  |  |  |  |
| Diabetes Duration (Months ± SD) |  | 29.8 ± 9.2 | 27.2 ± 7.0 | 27.7 ± 7.1 | NS |
|  |  |  |  |  |  |
| Induction |  |  |  |  | 0.70 |
| ATG and Thymoglobulin | 14 | 5 (22%) | 4 (19%) | 5 (42%) |  |
| Campath | 15 | 6 (26%) | 5 (24%) | 4 (33%) |  |
| Rituximab | 0 | 0 (0%) | 0 (0%) | 0 (0%) |  |
| IL-2 receptor antagonist | 27 | 12 (52%) | 12 (57%) | 3 (25%) |  |
|  |  |  |  |  |  |
| PRA (% Mean ± SD) |  | 3.27 ± 7.58 | 11.3 ± 30.3 | 2.00 ± 3.12 | 0.41 |

Supplemental Table 3: Recipients Characteristics for Primary Pancreas After Kidney (PAK) Transplants. Body Mass Index (BMI), Donation after Brain Death (DBD), Donation after Circulatory Death (DCD), Cold Ischemia Time (CIT), Standard Deviation (SD)

|  |  | **Nautical Miles Groups** | | | |
| --- | --- | --- | --- | --- | --- |
|  | Total | <250 NM | 250NM-750NM | >750NM | p-value |
| Age, years (Mean ± SD) |  | 41.5 ± 11.1 | 43.9 ± 10.6 | 43.2 ± 10.7 | 0.51 |
| BMI (Mean ± SD) |  | 26.9 ± 4.3 | 27.1 ± 3.6 | 26.1 ± 3.5 | 0.4 |
|  |  |  |  |  |  |
| Gender |  |  |  |  | 0.26 |
| Males (%) | 68 | 23 (38%) | 22 (55%) | 23 (44%) |  |
| Females (%) | 84 | 37 (62%) | 18 (45%) | 29 (56%) |  |
|  |  |  |  |  |  |
| Race |  |  |  |  | 0.46 |
| American Indian/Alaska Native | 0 | 0 (0%) | 0 (0%) | 0 (0%) |  |
| Asian | 1 | 1 (2%) | 0 (0%) | 0 (0%) |  |
| Black or African American | 0 | 0 (0%) | 0 (0%) | 0 (0%) |  |
| Native Hawaiian/Pacific Islander | 0 | 0 (0%) | 0 (0%) | 0 (0%) |  |
| White | 150 | 59 (98%) | 39 (98%) | 52 (100%) |  |
| Declines to answer | 1 | 0 (0%) | 1 (2%) | 0 (0%) |  |
|  |  |  |  |  |  |
| Indication for Transplant |  |  |  |  | NS |
| Diabetes Mellitus – Type I | 145 | 57 (95%) | 38 (95%) | 50 (96%) |  |
| Diabetes Mellitus – Type II | 0 | 0 (0%) | 0 (0%) | 0 (0%) |  |
| Diabetes Mellitus – Type Other/Unknown | 5 | 3 (5%) | 0 (0%) | 2 (4%) |  |
| Diabetes Secondary to chronic Pancreatitis | 2 | 0 (0%) | 2 (5%) | 0 (0%) |  |
|  |  |  |  |  |  |
| Diabetes Duration (Months ± SD) |  | 27.3 ± 11.0 | 26.4 ± 10.5 | 28.5 ± 10.6 | NS |
|  |  |  |  |  |  |
| Induction |  |  |  |  | 0.56 |
| ATG and Thymoglobulin | 71 | 28 (47%) | 18 (45%) | 25 (48%) |  |
| Campath | 49 | 19 (32%) | 16 (40%) | 14 (27%) |  |
| Rituximab | 0 | 0 (0%) | 0 (0%) | 0 (0%) |  |
| IL-2 receptor antagonist | 32 | 13 (22%) | 6 (15%) | 13 (25%) |  |
|  |  |  |  |  |  |
| PRA (% Mean ± SD) |  | 14.86 ± 28.0 | 13.8 ± 26.0 | 14.5 ± 26.8 | 0.93 |

Supplemental Table 4: Recipients Characteristics for Primary Pancreas Transplants Alone (PTA). Body Mass Index (BMI), Donation after Brain Death (DBD), Donation after Circulatory Death (DCD), Cold Ischemia Time (CIT), Standard Deviation (SD)
